# Human Tissue Kallikrein 1 Improves Erectile Dysfunction of Streptozotocin-Induced Diabetic Rats by Inhibition of Excessive Oxidative Stress and Activation of the PI3K/AKT/eNOS Pathway

**DOI:** 10.1155/2020/6834236

**Published:** 2020-02-28

**Authors:** Yang Luan, Kai Cui, Zhe Tang, Yajun Ruan, Kang Liu, Tao Wang, Zhong Chen, Shaogang Wang, Jihong Liu

**Affiliations:** Department of Urology, Tongji Hospital, Tongji Medical College, Huazhong University of Science and Technology, Wuhan 430030, China

## Abstract

**Objective:**

To investigate the protective effects and mechanisms of human tissue kallikrein 1 (hKLK1) on type 1 diabetes mellitus- (DM-) induced erectile dysfunction in rats. *Materials and Methods*. The homozygous transgenic rats (TGR) harboring the *hKLK1* gene and age-matched wild-type Sprague Dawley rats (WTR) were involved, and intraperitoneal injection of streptozotocin was utilized to induce diabetes in rats. Forty-eight-week-old male rats were randomly divided into a WTR group, TGR group, diabetic WTR group (WTDM), diabetic TGR group (TGDM), and TGDM with HOE140 group (TGDMH), with eight rats in each group. Twelve weeks later, the erectile response of all rats was detected by cavernous nerve electric stimulation, and corpus cavernosums were harvested to evaluate the levels of cavernous oxidative stress (OS), apoptosis, fibrosis, and involved pathways. Moreover, cavernous smooth muscle cells (CSMC) and endothelial cells (EC) were primarily isolated to build a coculture system for a series of *in vitro* verification.

**Results:**

The *hKLK1* gene and age-matched wild-type Sprague Dawley rats (WTR) were involved, and intraperitoneal injection of streptozotocin was utilized to induce diabetes in rats. Forty-eight-week-old male rats were randomly divided into a WTR group, TGR group, diabetic WTR group (WTDM), diabetic TGR group (TGDM), and TGDM with HOE140 group (TGDMH), with eight rats in each group. Twelve weeks later, the erectile response of all rats was detected by cavernous nerve electric stimulation, and corpus cavernosums were harvested to evaluate the levels of cavernous oxidative stress (OS), apoptosis, fibrosis, and involved pathways. Moreover, cavernous smooth muscle cells (CSMC) and endothelial cells (EC) were primarily isolated to build a coculture system for a series of

**Conclusions:**

hKLK1 preserves erectile function of DM rats through its antitissue excessive OS, apoptosis, and fibrosis effects, as well as activation of the PI3K/AKT/eNOS/cGMP pathway in the penis. Moreover, hKLK1 promotes relaxation and prevents high glucose-induced injuries of CSMC mediated by EC-CSMC crosstalk.

## 1. Introduction

According to the latest report from International Diabetes Federation, diabetes mellitus (DM) affects approximately 425 million people globally, and this number is expected to increase to 629 million by 2045. DM is one of the most common causes of penile erectile dysfunction (ED), characterized by the inability to develop or maintain the erection of penis during sexual intercourse, and 50%–75% of male DM patients suffer ED [1, 2]. DM can induce the accumulation of advanced glycation end products (AGEs) and a series of downstream oxidative stress (OS) reactions, followed by damage of endothelial function, apoptosis of functional cells, tissue morphological changes, and occurrence of multiple vascular system complications. DM-induced ED (DMED) is considered as the result of multiple pathogenetic factors, such as excessive cavernous OS, endothelial dysfunction, autonomic neuropathy, and penile fibrosis [1, 3, 4]. However, the first-line oral phosphodiesterase-5 (PDE5) inhibitors can neither increase cyclic guanosine monophosphate (cGMP) level if the bioavailability or formation of endogenous nitric oxide (NO) is restricted, nor reverse morphological changes of corpus cavernosum, which explains the poor responsiveness of DMED patients to PDE5 inhibitors [5]. Consequently, treatment towards single downstream target can hardly improve refractory DMED. Therefore, novel therapeutic methods are urgently needed for DMED.

Kininogens, kallikreins, and pharmacologically active kinins form the kallikrein-kinin system (KKS), which brings pleiotropic protection in many cardiovascular, cerebrovascular, and renal diseases [6, 7]. In the KKS, tissue kallikrein 1 (KLK1), a serine proteinase to be firstly discovered as a vasodilatory substance in human urine, locates on membrane of endothelial cell and can cleave kininogens, such as low-molecular-weight kininogen (LMWK), into several bioactive kinin peptides [6]. With respect to the *KLK1* gene delivery, some studies suggested its beneficial effects on inhibition of OS, apoptosis, fibrosis, and protection of endothelial cell [7, 8]. Moreover, our previous work found that the *hKLK1* gene present in transgenic rats (TGR) could play a preventive role in age-related ED through its multiple biological effects [9]. However, whether *hKLK1* can preserve erectile function at DM condition is not clear and worthy of further study.

Therefore, we firstly established the DM rat model in wild-type Sprague Dawley (SD) rats (WTR) and TGR through injection of streptozotocin (STZ), and then explored the detail effects and protective mechanisms of hKLK1 on DMED *in vivo*. Meanwhile, we primarily isolated and cocultured the endothelial cells (EC) and cavernous smooth muscle cells (CSMC), the principle cells to control erection, to determine the potential signaling pathway and cellular protective effects of hKLK1 involved in normal penile erection and abominable high-glucose condition *in vitro*. Our results provided an experimental basis for the therapeutic effects of hKLK1 as a promising alternative target for DMED.

## 2. Materials and Methods

### 2.1. Acquisition of the TGR

We obtained TGR harboring the *hKLK1* gene as a generous gift from the Max Delbrück Center for Molecular Medicine (Berlin, Germany). The TGR were generated by microinjecting a 5.6 kb DNA fragment containing the entire *hKLK1* gene, under the control of heavy metal-responsive mouse metallothionein promoter, into the oocytes of SD rats. The presence of the transgene in genomic DNA was verified by Southern blot [8]. Offsprings with homozygous *hKLK1* gene were selected for further experiments.

### 2.2. Verification of hKLK1 in TGR and EC

To verify the *hKLK1* gene in TGR, agarose gel electrophoresis following conventional polymerase chain reaction (PCR), real-time reverse transcriptase PCR (RT-PCR) and western blot were used to determine its existence and expression in frozen penile tissue samples. Bradykinin, the catalytic product of hKLK1, in penile tissue was detected by enzyme-linked immunosorbent assay (ELISA). The expression of hKLK1 in EC was detected by immunofluorescence.

### 2.3. Isolation, Culture, Identification, and Coculture of CSMC and Aorta EC

As described before [10], the CSMC were primarily isolated from penile cavernosum by tissue attached method and cultured in Dulbecco's modified Eagle's medium (DMEM, Hyclone, USA) containing 10% fetal bovine serum (FBS) at 37 °C in an atmosphere containing 5% CO_2_. Expressions of desmin were selected for the identification of CSMC by immunofluorescence. As penile vessels are parts of systematic circulation and cavernous EC is difficult to purify with ideal viability for following experiments, we selected aorta EC with similar shape and function instead [11, 12]. Aorta of rat was isolated and digested by collagenase I (Invitrogen, USA) and then minced into 5 mm strips adhere to Matrigel (BD Biosciences, USA) and soaked in Endothelial Growth Medium-2 with endothelial cell growth factor (EGM2, Lonza Biologics, USA) [13]. After five-day incubation, EC were further cultured with tissues pieces removed. Expression of CD31 in EC was identified by immunofluorescence.

The coculture system was established by a Transwell system (24 wells, 0.4 *μ*m pore size). The upper chamber was seeded with CSMC in DMEM. After the attachment of CSMC, the aorta EC were then seeded onto the lower chamber with EGM2. All following *in vitro* experiments were based on this coculture system.

### 2.4. Immunofluorescence

Cells were prepared and detected as showed in previous study [14]. Primary antibodies against hKLK1 (1 : 200; Sigma-Aldrich), CD31 (1 : 100, Boster), and desmin (1 : 200; Abcam) were used in this study. Nuclei were stained with diamidino-2-phenylindole (DAPI, Boster).

### 2.5. NO Detection

Penile tissues were prepared according to the manufacturer's protocol of the nitrate-nitrite assay kit (Beyotime Biotechnology). The total NO levels were detected at the peak absorbance of A540 nm with the classic Griess Reagent. The results were standardized by their protein concentration.

### 2.6. Detection of cGMP and Ca^2+^

*In vitro*, 10 nM LMWK was used to treat EC to activate hKLK1. 1 *μ*M HOE140, 10 *μ*M LY294002, and 100 *μ*M L-NAME were used to treat CSMC to, respectively, suppress bradykinin receptor 2 (B_2_R), PI3K, and eNOS. Seven groups, including WTR, WTR+LMWK, TGR, TGR+LMWK, TGR+LMWK+HOE140, TGR+LMWK+LY294002, and TGR+LMWK+L-NAME, were set. After incubation of agents for 30 min, CSMC were collected for cGMP concentration detection with an ELISA kit (Westang). Similarly, the detection of Ca^2+^ concentration in CSMC were performed with fluorescent probe according to the manufacturer's instructions (Beyotime Biotechnology). Additionally, CSMC in groups of WTR+LMWK, TGR+LMWK, and TGR+LMWK+HOE140 were incubated in DMEM with different concentration of glucose (5 nM and 30 nM) for three days. Then, we tested the cGMP concentration in CSMC by ELISA method.

*In vivo*, the cGMP concentration in penile tissues were also detected by ELISA kit as described above.

### 2.7. Real-Time RT-PCR

We harvested penile tissues and extracted its total RNA using the Multisource Total RNA Minipre Kit (Axygen). Real-time RT-PCR was conducted with the PrimeScript RT Master Mix and SYBR Green PCR Master Mix (TaKaRa). The mRNA expressions of certain genes relative to *β*-actin were calculated using the 2^−△△Ct^ method. The primer sequences of *hKLK1*, rat *KLK1* (*rKLK1*), and *β*-actin are shown in Table 1.

### 2.8. Western Blot Analysis

As showed in pervious study [9], relative proteins expressions of CSMC from coculture system and rats' penile tissues were determined through western blot method. The primary antibodies used included anti-hKLK1 (1 : 5,000; Sigma-Aldrich), anti-rKLK1 (1 : 1,000; Sigma-Aldrich), anti-advanced glycation end products receptor (RAGE; 1 : 1,000; Abcam), anti-NOX2 (1 : 1,000; Affinity), anti-P67^phox^ (1 : 1,000; Affinity), anti-P47^phox^ (1 : 1,000; Affinity), anti-Caspase3 (1 : 1,000; Proteintech), anti-cleaved Caspase3 (1 : 1,000; Proteintech), anti-B-cell lymphoma-2 (Bcl-2; 1 : 1,000; Proteintech), anti-Bcl-2-associated X protein (Bax; 1 : 1,000; Affinity), anti-phosphatidylinositol 3-kinase (PI3K(p55), 1 : 1,000; Cell Signaling Technology), anti-protein kinase B (AKT; 1 : 1000; CST), anti-phospho-AKT (pAKT, Ser473, 1 : 1000; CST), anti-eNOS (1 : 1,000; Abcam), anti-phospho-eNOS(Ser1177) (PeNOS(S1177), 1 : 1,000; Abcam), anti-phospho-eNOS(Thr495) (PeNOS(T495), 1 : 1,000; Abcam), anti-PDE5 (1 : 1,000; Abcam), anti-ICa-L (1 : 1,000; Abcam), anti-transforming growth factor-*β*1 (TGF*β*1; 1 : 1,000; Abcam), anti-Smad2/3 (1 : 1,000; CST), anti-phospho-Smad2/3 (1 : 1,000; CST), anti-CTGF (1 : 1,000; Abcam), anti-collagen I (1 : 1,000; Proteintech), anti-collagen IV (1 : 1,000; Proteintech), and anti-*β*-actin (1 : 1,000; Proteintech). Membranes were then incubated with horseradish peroxidase-conjugated secondary antibodies (1 : 5,000; Proteintech) and analyzed using an enhanced chemiluminescence detection system (Thermo Fisher Scientific).

### 2.9. Histological Analysis

Penile tissues sections (5 *μ*m thickness) were incubated with antibodies against CD31 (1 : 100; Abcam), *α*-SMA (1 : 200; Abcam), and TGF*β*1 (1 : 100; Abcam) overnight at 4 °C. Sections were then washed three times and incubated with a biotinylated secondary antibody (1 : 1,000; Proteintech). Finally, antigen-antibody reactions were detected by staining with diaminobenzidine (Beyotime Biotechnology). Images were captured at ×100 magnification and semiquantitative analysis were performed to evaluate staining intensity using ImagePro Plus software (Media Cybernetics).

Smooth muscle and collagen content were quantitatively analyzed in Masson's trichrome-stained sections. The ratio of smooth muscle area (red) to collagen area (blue) were considered to reflect tissue fibrosis. These data were calculated using ImagePro Plus software (Media Cybernetics) in five randomly selected sections per group.

### 2.10. OS and Apoptosis Level Detection

CSMC in WTR and TGR coculture systems were cultured in DMEM with different glucose concentration (5 nM and 30 nM) for three and seven days. We detected reactive oxygen species (ROS) activity by fluorescence probe method according to the protocol of test kit (Beyotime Biotechnology). OS in corpus cavernosum was evaluated by levels of an oxidant, malondialdehyde (MDA), and an antioxidant, superoxide dismutase (SOD), with test kits (Beyotime Biotechnology). Penile MDA level and SOD activity were normalized by the wet weight of penile tissue samples.

The apoptosis level of CSMC in coculture system were tested through flow cytometry according to the standard protocol (BD Biosciences). Apoptotic cells in penile sections were stained using an In Situ Cell Death Detection kit (Roche Applied Science). Apoptotic index (AI), the percentage of apoptotic cells, were calculated to represent apoptosis level.

### 2.11. Animals

Experiments were approved by the Institutional Animal Care and Use Committee at the Tongji Hospital, Tongji Medical College, Huazhong University of Science and Technology. 16 eight-week WTR and 24 age-matched TGR were used in this study. WTR were randomly divided into two groups: the normal Control (WTR group; *n* = 8) and the DMED model (WTDM group; *n* = 8). TGR were randomly divided into the TGR group (*n* = 8), the transgenic diabetic rats group (TGDM; *n* = 8), and the transgenic diabetic rats treated with HOE140 group (TGDMH; *n* = 8). Rats in the WTDM, TGDM, and TGDMH groups were intraperitoneally injected with streptozotocin (STZ, 40 mg/kg, Sigma-Aldrich) dissolved in citrate buffer solution (0.1 mol/L, pH = 4.5), while rats in the WTR and TGR groups were injected with equal amount of 0.1 mol/L citric acid-sodium citrate buffer solution instead. After 72 hours, only rats with fasting glucose concentrations higher than 16.7 mmol/L were considered to have DM. In addition, rats in the TGDMH group were also intraperitoneally injected with HOE140 (10 *μ*g/kg/d; Sigma-Aldrich), a specific inhibitor of B_2_R to suppress hKLK1 signaling, after confirmation of DM. 12 weeks later, we recorded the final metabolic parameters and conducted erectile function test.

### 2.12. Erectile Function Test

The intracavernous pressure (ICP) was measured as previously described [9]. Briefly, rats were anesthetized, and cavernous nerve were exposed. A 25-gauge butterfly needle was inserted into the left penile crus to monitor ICP. Then, a PE-50 tube filled with heparinized saline (200 IU/mL) was cannulated into the left common carotid artery and connected to a pressure transducer (PowerLab 4SP; AD Instruments) to continuously monitor arterial blood pressure (ABP). Electric stimulation parameters were 2.5, 5.0, and 7.5 votes, 15 Hz, pulse width of 1.2 microsecond, with duration of one minute. The ratios of the peak ICP to mean ABP (MAP) (peak ICP/MAP) and the area under the curve (AUC) to MAP were calculated to evaluate their erectile function.

### 2.13. Statistical Analysis

Each result came from at least three independent experiments. Data are presented as mean ± standard error of the mean (SEM). Statistical significance was assessed using Student's *t*-test for comparisons of two groups or one-way analysis of variance followed by a *post hoc* (Tukey) test for comparisons between multiple groups. *P* < 0.05 was considered statistically significant.

## 3. Results

### 3.1. Metabolic Parameters

As shown in Table 2, the initial body weight and fasting blood glucose among all groups have no significant difference (*P* > 0.05). However, at 12 weeks, rats in WTDM, TGDM, and TGDMH groups had higher final body weight and fasting blood glucose levels than those in WTR and TGR groups (all *P* < 0.001). These metabolic variables suggested the successful establishment of rat DM model.

### 3.2. Verification of TGR and Cell Types

**Figure 1:**
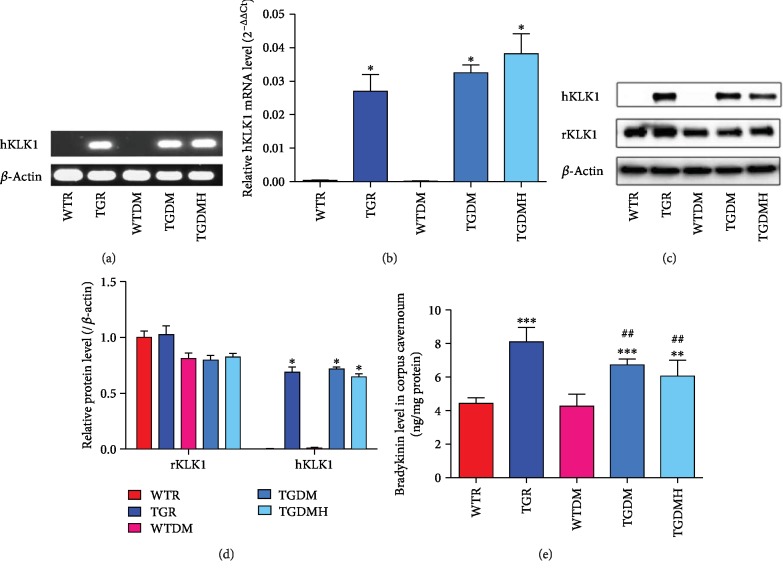
Verification of the existence and expression of *hKLK1* and *rKLK1* genes in the corpus cavernosum. Panel (a) shows representative *hKLK1* genomic DNA bands in the corpus cavernosum through conventional polymerase chain reaction followed by agarose gel electrophoresis. Panel (b) shows relative mRNA expression of the hKLK1 to *β*-actin within the corpus cavernosum of different groups by real-time reverse transcriptase polymerase chain reaction. Data are shown as mean ± standard error of the mean with six samples per group. Panels (c) and (d) show rKLK1 and hKLK1 protein expressions in the corpus cavernosum by western blot with five samples per group. Panel (e) shows bradykinin level in all groups. Each bar represents mean ± standard error of the mean. ^∗^*P* < 0.05, ^∗∗^*P* < 0.01, and ^∗∗∗^*P* < 0.001 compared to WTR group. ^##^*P* < 0.01 compared to WTDM group. hKLK1: human tissue kallikrein 1; rKLK1: rat tissue kallikrein 1; WTR: wild-type rats; TGR: transgenic rats; WTDM: diabetic wild-type rats; TGDM: diabetic transgenic rats; TGDMH: diabetic transgenic rats administrated with HOE140.

All rats in TGR, TGDM, and TGDMH groups contained the *hKLK1* gene and were able to transcribe and translate them into hKLK1 mRNA and protein, while *hKLK1* gene did not exist in rats of WTR and WTDM groups. In addition, there was no obvious difference of rKLK1 expression among all groups (Figure 1).

**Figure 2:**
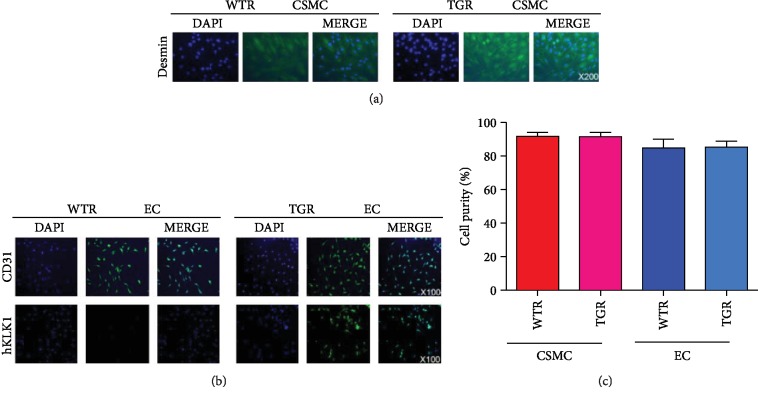
Verification of CSMC and EC from WTR and TGR and the expression of the hKLK1 in EC by immunofluorescence. Panel (a) shows the verification of CSMC through the expression of desmin (magnification: ×200). Panel (b) shows the verification of EC through the expression of CD31, and hKLK1 was only expressed in EC from TGR but not WTR (magnification: ×100). Panel (c) shows the cell purities of EC and CSMC from WTR and TGR through the bar graph. hKLK1: human tissue kallikrein 1; rKLK1: rat tissue kallikrein 1; WTR: wild-type rats; TGR: transgenic rats; EC: endothelial cell; CSMC: cavernous smooth muscle cell; DAPI: 4′,6-diamidino-2-phenylindole.

We determined the expression of desmin in CSMC and CD31, hKLK1 in EC. Our data suggested the CSMC and EC were successfully isolated with high purity. Only EC from TGR, but not WTR, expressed hKLK1 protein (Figure 2).

### 3.3. hKLK1 Preserved Erectile Function in DM Rats

**Figure 3:**
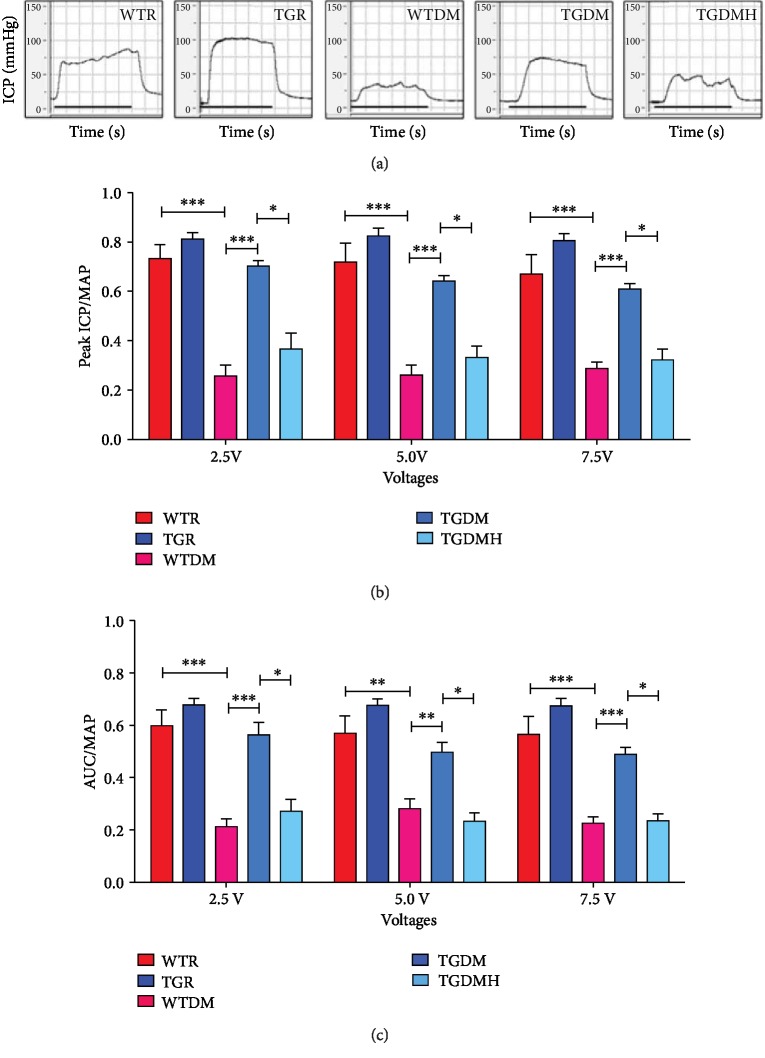
Erectile response of all rats was evaluated by cavernous nerve electrostimulation. Panel (a) shows representative curves of ICP at 5.0 V. Panels (b) and (c) show the ratios of peak ICP to MAP and total ICP (AUC) to MAP in relation to penile erectile capacity in each group at 2.5 V, 5.0 V, and 7.5 V. Each bar represents mean ± standard error of the mean. ^∗^*P* < 0.05, ^∗∗^*P* < 0.01, and ^∗∗∗^*P* < 0.001. WTR: wild-type rats; TGR: transgenic rats; WTDM: diabetic wild-type rats; TGDM: diabetic transgenic rats; TGDMH: diabetic transgenic rats administrated with HOE140; ICP: intracavernous pressure; AUC: area under the curve.

As illustrated in Figure 3, the WTR group exhibited a normal ICP curve and high ratios of peak ICP/MAP and AUC/MAP at electrostimulation of 5.0 V (0.72 ± 0.078 and 0.57 ± 0.066). The TGR group had similar levels (0.83 ± 0.033 and 0.68 ± 0.020) without significant difference. WTDM group had much lower ratios (0.26 ± 0.042 and 0.28 ± 0.038, *P* < 0.01) than WTR group. Fortunately, erectile function was partly preserved in the TGDM group (0.64 ± 0.024 and 0.50 ± 0.038, *P* < 0.01) than those in the WTDM group, although it was still lower than WTR and TGR groups. However, TGDMH group (0.33 ± 0.046 and 0.23 ± 0.032, *P* < 0.01) had lower ICP curve and ratios of peak ICP/MAP and AUC/MAP than TGDM group. We also recorded those pressure curves and calculated the ratios of peak ICP/MAP and AUC/MAP at 2.5 V and 7.5 V, which presented same tendency as those at 5.0 V (shown in Figure 3).

### 3.4. hKLK1 Inhibited OS and Apoptosis *In Vivo*

**Figure 4:**
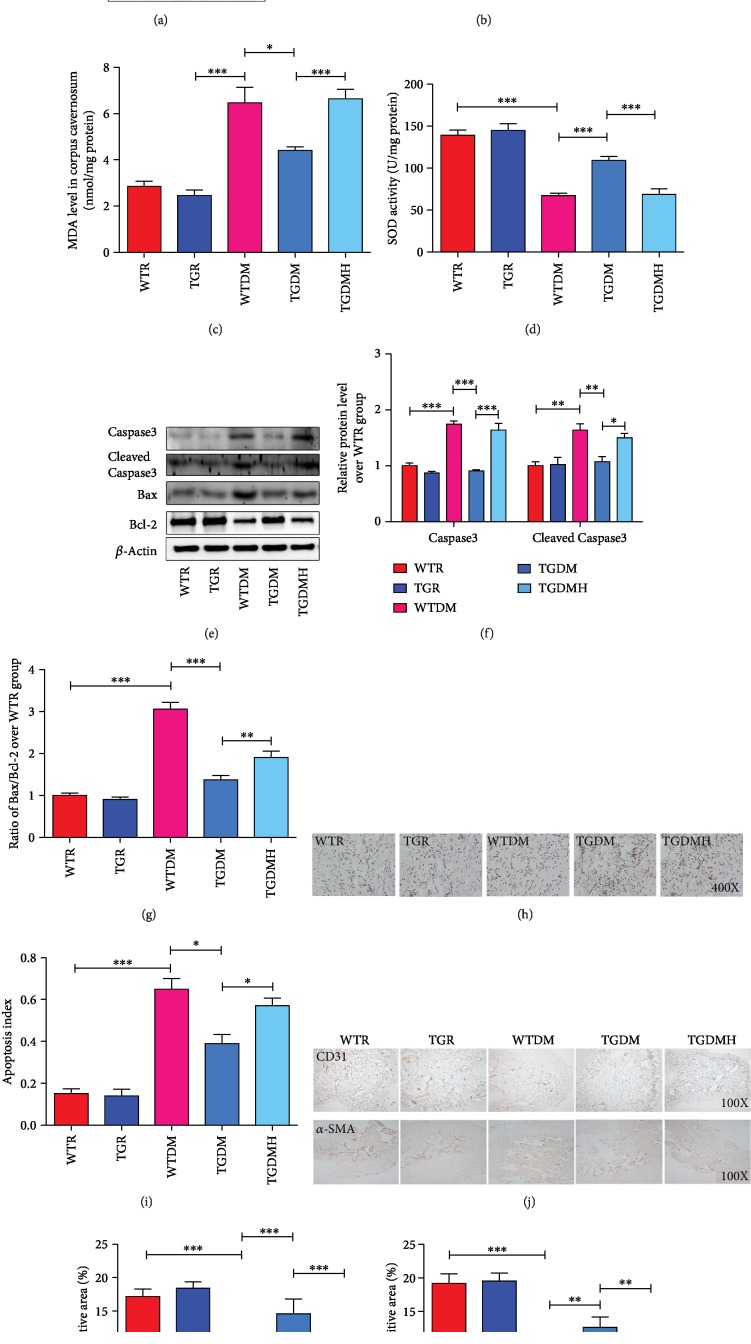
hKLK1 could inhibit oxidative stress and apoptosis in the corpus cavernosum of diabetic rats. Panels (a) and (b) show protein expressions of RAGE, NOX2, p67^phox^, and p47^phox^ normalized to *β*-actin level. Panels (c) and (d) show MDA level and SOD activity normalized to total protein concentration of penile samples. Panels (e) and (f) show protein expressions of Caspase3 and cleaved Caspase3 normalized to *β*-actin level. Panel (g) shows the ratio of Bax/Bcl-2 in different groups. Apoptosis levels were assayed by the terminal deoxynucleotidyl transferase-mediated nick end labeling method (h, magnification: ×400). Panel (i) shows the calculated apoptosis index (percentage of apoptotic cells [brown stained] to all stained cells), which was used to quantify cavernous apoptosis level. Panel (j) shows the expressions of CD31 and *α*-SMA through immunohistochemical staining in all groups of penile tissues (magnification: ×100). Panels (k) and (l) show the positive area (%) of CD31 and *α*-SMA, respectively. Each bar represents mean ± standard error of the mean. ^∗^*P* < 0.05, ^∗∗^*P* < 0.01, and ^∗∗∗^*P* < 0.001. hKLK1: human tissue kallikrein 1; MDA: malondialdehyde; SOD: superoxide dismutase. WTR: wild-type rats; TGR: transgenic rats; WTDM: diabetic wild-type rats; TGDM: diabetic transgenic rats; TGDMH: diabetic transgenic rats administrated with HOE140.

The RAGE and NADPH oxidase, including several subunits such as p47^phox^, NOX2, and p67^phox^, are regarded as key molecules in the progression of DM complications. As showed in Figures 4(a) and 4(b), the expressions of RAGE and p47^phox^ of the TGR group were lower than those of the WTR group (*P* < 0.05), while the expressions of NOX2 and p67^phox^ had no difference between the WTR and TGR groups (*P* > 0.05). Compared to WTR group, the expressions of RAGE, NOX2, p47^phox^, and p67^phox^ of WTDM group increased sharply (*P* < 0.01), and hKLK1 in TGDM group could inhibited their levels (*P* < 0.01). However, they were upregulated again in TGDMH group (*P* < 0.05). MDA levels showed a similar trend as NOX2 (Figure 4(c); all *P* < 0.05). Contrarily, SOD, an antioxidant, presented reverse activity changes as MDA (Figure 4(d); all *P* < 0.001).

We also tested the apoptosis level through western blot and TUNEL methods. The expressions of Caspase3, cleaved Caspase3, Bax, and Bcl-2 showed no difference between WTR and TGR groups (*P* > 0.05). In WTDM group, the ratio of Bax/Bcl-2 and the expression of Caspase3 and cleaved Caspase3 were higher than those in WTR group (*P* < 0.001). However, they were all reduced in TGDM group (*P* < 0.001), and increased again in TGDMH group (Figures 4(e)–4(g); *P* < 0.01). Similarly, the apoptosis index in WTDM was more than three times than control group, and hKLK1 could much improve it (Figures 4(h) and 4(i); all *P* < 0.05). In accordance with the apoptosis result, immunohistochemical results of CD31 and *α*-SMA protein expressions, representing the content changes of cavernous endothelium and smooth muscle, in corpus cavernosum slides showed sharp decrease in WTDM group than the control group. But they were increased in TGDM group, and these improvement could be diminished by HOE140 (Figures 4(j)–4(l)).

### 3.5. hKLK1 Preserved the PI3K/AKT/eNOS Signaling Activity in DM Rats

**Figure 5:**
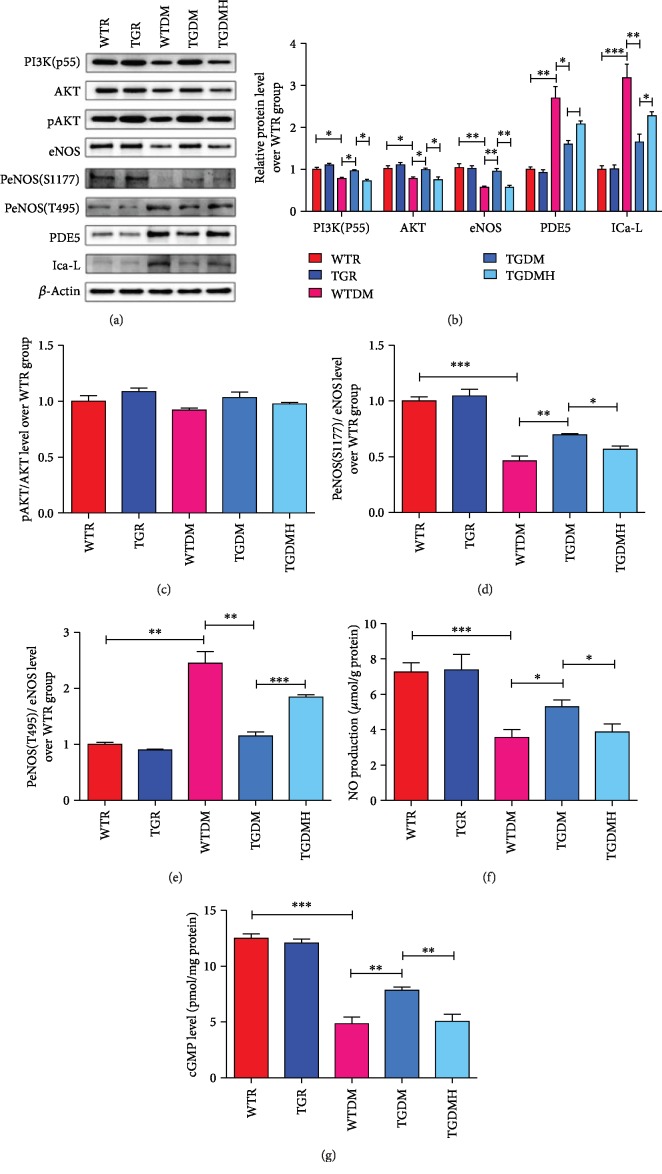
hKLK1 could activate PI3K/AKT/eNOS signaling in the corpus cavernosum of diabetic rats. Panels (a) and (b) show protein expressions of PI3K(p55), PDE5, and ICa-L normalized to *β*-actin level, and panels (c), (d), and (e) show the ratios of pAKT/AKT, PeNOS(S1177)/eNOS, and PeNOS(T495)/eNOS to evaluate their activity in all groups. Panels (f) and (g) show NO and cGMP levels normalized to total protein concentration of penile samples. Each bar represents mean ± standard error of the mean. ^∗^*P* < 0.05, ^∗∗^*P* < 0.01, and ^∗∗∗^*P* < 0.001. hKLK1: human tissue kallikrein 1; NO: nitric oxide; cGMP: cyclic guanosine monophosphate; WTR: wild-type rats; TGR: transgenic rats; WTDM: diabetic wild-type rats; TGDM: diabetic transgenic rats; TGDMH: diabetic transgenic rats administrated with HOE140.

In Figure 5, we found that the expression of PI3K(p55), AKT, and eNOS and the phosphorylation of AKT and eNOS(Ser1177) of WTDM group were lower, while the expressions of phosphorylated eNOS(Thr495), PDE5, and ICa-L were higher when compared to WTR group. These changes were all improved significantly in TGDM group, while they were deteriorated again in TGDMH group (Figures 5(a)–5(e); all *P* < 0.05). In addition, the NO and cGMP levels in WTDM group dropped sharply when compared to WTR group. However, they were upregulated in TGDM group, while got suppressed by HOE140 (Figures 5(f) and 5(g); all *P* < 0.05).

### 3.6. hKLK1 Inhibited Penile Morphological Changes in DM Rats

**Figure 6:**
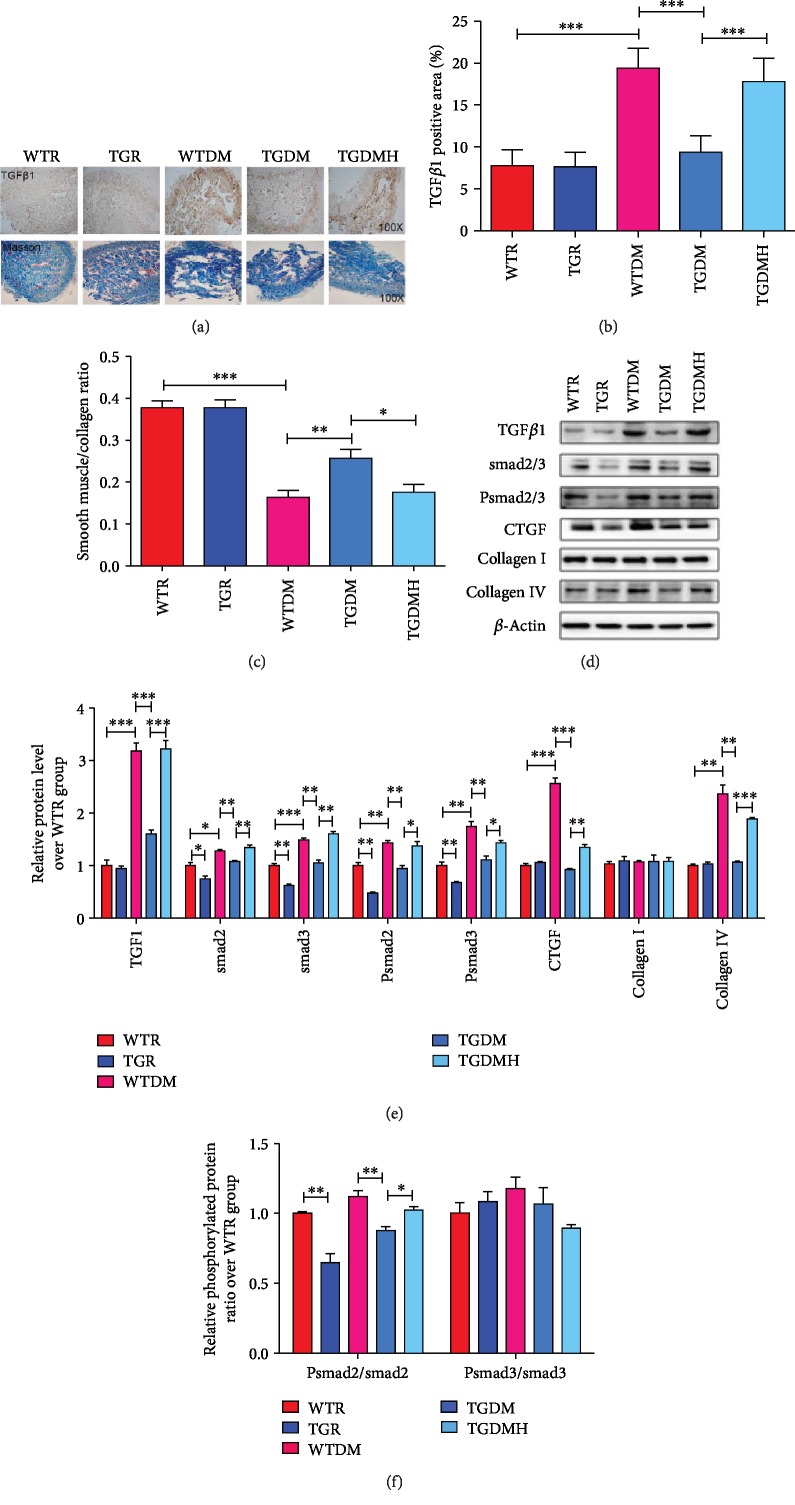
hKLK1 could inhibit cavernous fibrosis in diabetic rats. Histologic features were evaluated by the expression and location of TGF*β*1 and Masson trichrome stain (a, magnification: ×100). The positive area (%) of TGF*β*1 (b) and the ratio of smooth muscle (red stain) to collagen (blue stain) contents in each penile section (c) were calculated to show the degree of cavernous fibrosis. Panels (d) and (e) show protein expressions of TGF*β*1, smad2/3, Psmad2/3, CTGF, collagen I, and collagen IV normalized to *β*-actin level. The ratios of Psmad2/smad2 and Psmad3/smad3 were calculated to evaluate their activity in all groups (f). Each bar represents mean ± standard error of the mean. ^∗^*P* < 0.05, ^∗∗^*P* < 0.01, and ^∗∗∗^*P* < 0.001. hKLK1: human tissue kallikrein 1; TGF*β*1: transforming growth factor-*β*1; CTGF: connective tissue growth factor; WTR: wild-type rats; TGR: transgenic rats; WTDM: diabetic wild-type rats; TGDM: diabetic transgenic rats; TGDMH: diabetic transgenic rats administrated with HOE140.

Penile morphological changes were evaluated by immunohistochemical staining and western blot. When compared to WTR group, the TGF*β*1 expression in slide of WTDM group was higher, while the change was inhibited in the TGDM group, and reelevated in the TGDMH group. In consistent with this result, Masson's staining showed the ratio of smooth muscle/collagen of WTDM group decreased distinctly compared to WTR group, while it was improved in TGDM group. However, it got lower in TGDMH group again (Figures 6(a)–6(c)). Moreover, through western blot, the expressions of TGF*β*1, connective tissue growth factor (CTGF), ratio of Psmad2/smad2, and collagen IV in WTDM group were significantly higher than those in WTR group. They were inhibited clearly by hKLK1 in the TGDM group and increased again by HOE140 (all *P* < 0.05). In addition, there were no evident difference for the ratio of Psmad3/smad3 and collagen I expression among five groups (Figures 6(d)–6(f)).

### 3.7. hKLK1 Preserved the cGMP Level and Reduced Ca^2+^ Concentration in CSMC

**Figure 7:**
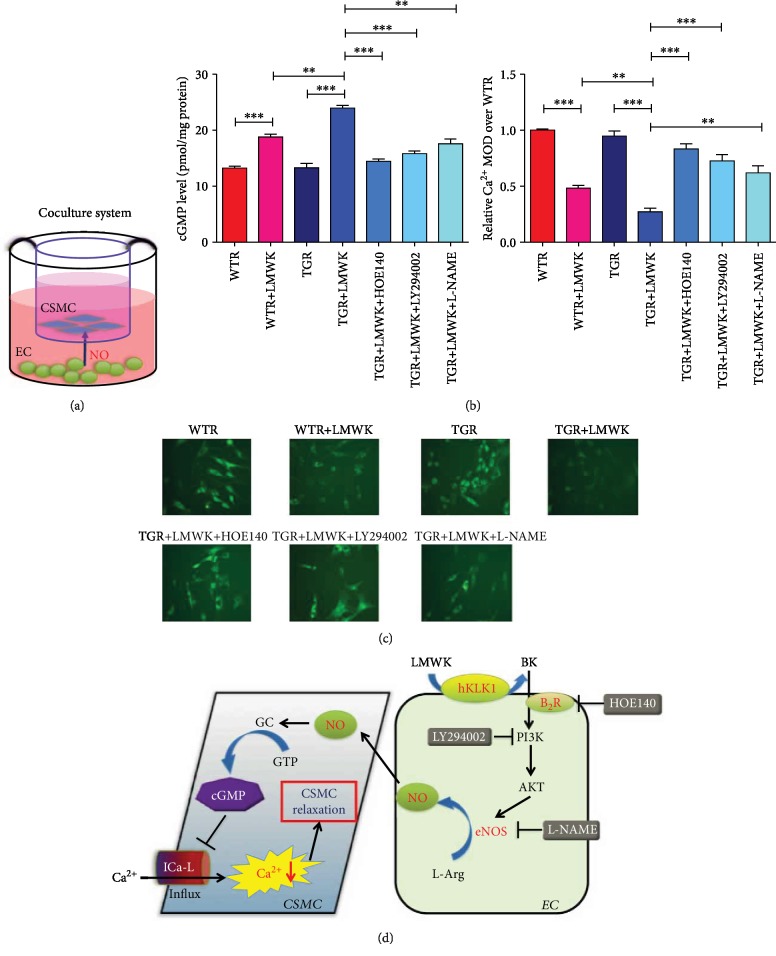
hKLK1 promoted cGMP production and inhibited Ca^2+^ concentration in CSMC by the PI3K/AKT/eNOS pathway under physiological condition. The diagram of the coculture system of CSMC and EC is shown in panel (a). Panels (b) and (c) show the cGMP and intracellular Ca^2+^ level changes at the administration of LMWK, HOE140, LY294002, and L-NAME in both CSMC from WTR and TGR. All images were captured at magnification of 200x. Panel (d) shows the potential signaling pathway downstream to hKLK1 involved in normal CSMC relaxation. Each bar represents mean ± standard error of the mean. ^∗^*P* < 0.05, ^∗∗^*P* < 0.01, and ^∗∗∗^*P* < 0.001. hKLK1: human tissue kallikrein 1; NO: nitric oxide; cGMP: cyclic guanosine monophosphate; CSMC: cavernous smooth muscle cell; EC: endothelial cells; WTR: wild-type rats; TGR: transgenic rats; WTDM: diabetic wild-type rats; TGDM: diabetic transgenic rats; TGDMH: diabetic transgenic rats administrated with HOE140.

The diagram of coculture system is showed in Figure 7(a). Physiologically, the cGMP levels in WTR+LMWK and TGR+LMWK groups (18.77 ± 0.524 and 23.97 ± 0.484) were higher than WTR and TGR groups, respectively (13.23 ± 0.318 and 13.27 ± 0.784; both *P* < 0.001). Meanwhile, the cGMP level of TGR+LMWK group was significantly higher than WTR+LMWK group and were dramatically dropped down after adding HOE140, LY294002, and L-NAME to block B_2_R, PI3K, and eNOS (Figure 7(b); all *P* < 0.01).

Parallel to the results of cGMP, the intracellular Ca^2+^ concentration in CSMC of WTR+LMWK and TGR+LMWK groups were lower than WTR and TGR groups, respectively (both *P* < 0.001). And Ca^2+^ level was also lower in TGR+LMWK group than WTR+LMWK group (*P* < 0.001). Moreover, the Ca^2+^ concentration in TGR+LMWK+HOE140, TGR+LMWK+LY294002, and TGR+LMWK+L-NAME groups were greatly higher than TGR+LMWK group (Figure 7(c); all *P* < 0.01). However, there was no difference between WTR and TGR groups without adding the ligand of hKLK1. These data demonstrated the potential downstream B_2_R/PI3K/eNOS/cGMP signaling pathway of hKLK1 on CSMC relaxation (Figure 7(d)).

### 3.8. hKLK1 Inhibited OS, Apoptosis, and cGMP Level Induced by High Glucose in CSMC

**Figure 8:**
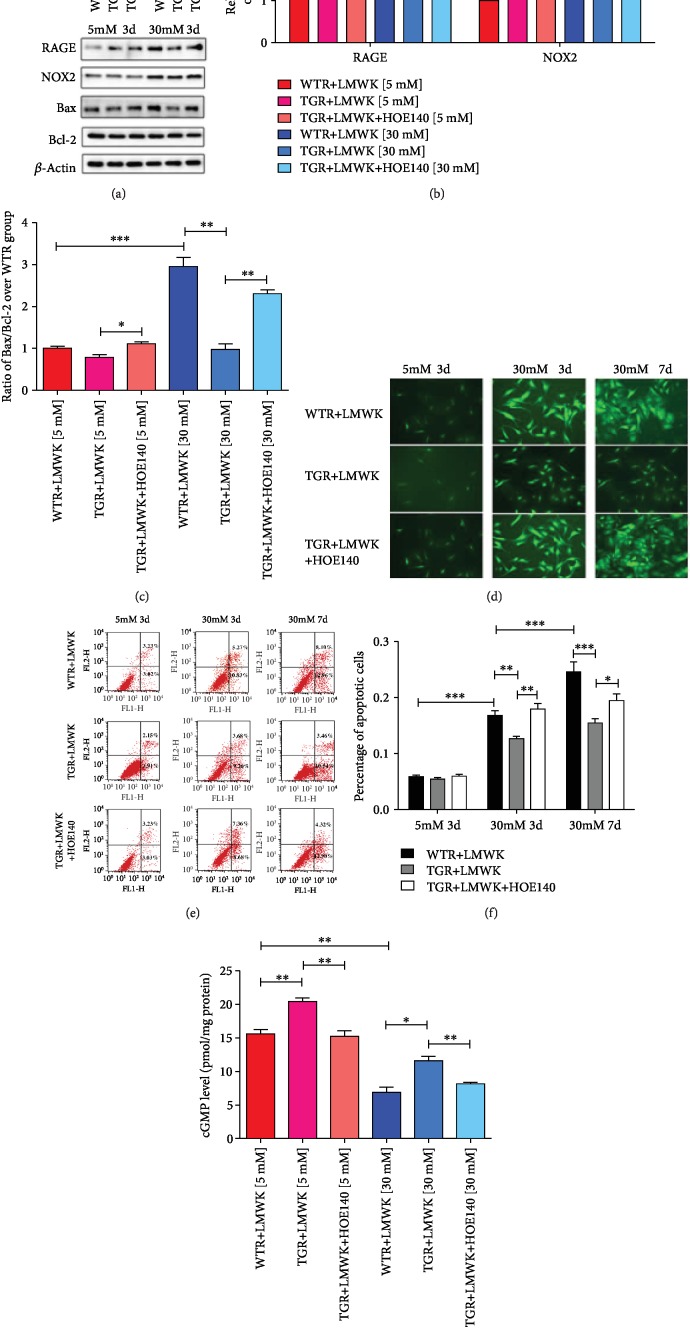
hKLK1 inhibited oxidative stress, cell apoptosis, and cGMP production in CSMC under high-glucose condition. Panels (a), (b), and (c) show protein expressions of RAGE, NOX2, Bax, and Bcl-2 level normalized to *β*-actin and Bax/Bcl-2 ratio in CSMC under normal and high-glucose culture conditions. Panels (d) to (f) show the ROS production and percentage of apoptotic CSMC under different glucose concentrations and incubation times by fluorescence and flow cytometry in WTR and TGR with or without HOE140. All images were captured at magnification of 200x. Panel (g) shows cGMP level under normal and high-glucose culture conditions. Each bar represents mean ± standard error of the mean. ^∗^*P* < 0.05, ^∗∗^*P* < 0.01, and ^∗∗∗^*P* < 0.001. hKLK1: human tissue kallikrein 1; ROS: reactive oxygen species. WTR: wild-type rats; TGR: transgenic rats; LMWK: low-molecular-weight kininogen; BK: bradykinin; PI3K: phosphatidylinositol 3-kinase; AKT: protein kinase B; eNOS: endothelial nitric oxide synthase; NO: nitric oxide; cGMP: cyclic guanosine monophosphate; GTP: guanosine triphosphate; GC: guanylate cyclase; EC: endothelial cell; CSMC: cavernous smooth muscle cell.

As showed in Figure 8, hKLK1 had no impact on the level of OS and apoptosis in CSMC at normal culture condition. However, high-glucose condition induced high expressions of RAGE, NOX2, and ROS, revealing increased level of OS, in CSMC. Although hKLK1 in TGR+LMWK group downregulated those index above, HOE140 could weaken its anti-OS effect (Figures 8(a), 8(b), and 8(d)). Parallel to OS evaluation, hKLK1 could resist high glucose-induced increased level of Bax/Bcl-2 ratio and percentage of apoptotic CSMC. This antiapoptosis effect could also be erased by hKLK1 pathway inhibitor (Figures 8(c), 8(e), and 8(f)). In terms of cGMP production in CSMC, activating hKLK1 pathway could increase cGMP level at physiological status and high glucose could suppress it to half level of control group. Fortunately, hKLK1 could still improve its level at high-glucose condition (Figure 8(g)).

## 4. Discussion

**Figure 9:**
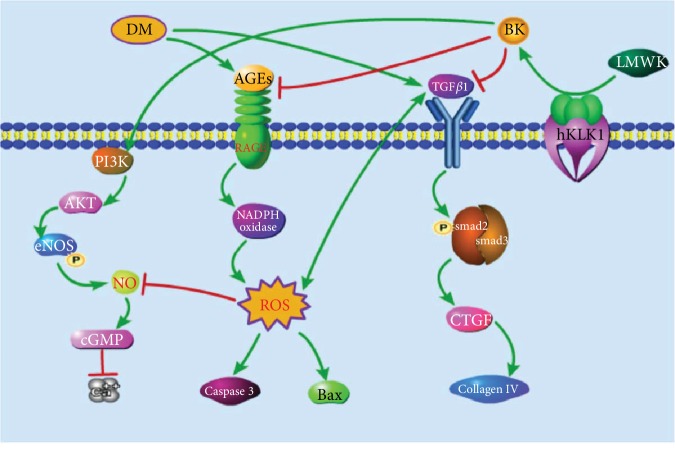
The potential mechanisms of hKLK1's protective effects on DMED. DM can activate RAGE/NADPH oxidase signaling to induce the generation of ROS. Subsequently, ROS promotes the apoptosis-related protein expression including Bax and cleaved Caspase3, activates the TGF*β*1/smad2/3/CTGF signaling to induce the collagen IV expression, and inhibits the NO/cGMP pathway to increase the intracellular Ca^2+^ level in CSMC. By the catalysis of hKLK1, LMWK can be converted into BK and then inhibits RAGE/ROS and TGF*β*1 expression and activates the PI3K/AKT/eNOS pathway to improve oxidative stress, apoptosis, fibrosis, and CSMC relaxation. hKLK1: human tissue kallikrein 1; DM: diabetes mellitus; DMED: DM-induced erectile dysfunction; BK: bradykinin; RAGE: receptor for advanced glycation end products; ROS: reactive oxygen species. LMWK: low-molecular-weight kininogen; BK: bradykinin; Bax: Bcl-2-associated X protein; PI3K: phosphatidylinositol 3-kinase; AKT: protein kinase B; eNOS: endothelial nitric oxide synthase; NO: nitric oxide; cGMP: cyclic guanosine monophosphate; TGF*β*1: transforming growth factor-*β*1; CTGF: connective tissue growth factor; CSMC: cavernous smooth muscle cell.

Here, through transgenic rats harboring the *hKLK1* gene, we initially demonstrated that hKLK1 could preserve the erectile function in DM rats by inhibition of excessive OS and apoptosis, correction of cavernous histologic abnormalities, and activation of PI3K/AKT/eNOS signaling to generate more cGMP *in vivo* and *in vitro* (shown in Figure 9).

ED is one of earliest and frequent complications of DM, which affects the normal quality of life and presages underlying vasculopathy and serious cardiovascular diseases [1]. 12 weeks after establishment of STZ-induced DM rat model, we found that DM seriously impaired the normal erectile response, while the *hKLK1* could obviously preserve it, and this protective effect could be diminished by inhibitor of hKLK1 pathway.

OS is characterized by an imbalance between the OS production by prooxidants and the clearance of excess ROS by antioxidants at the cellular level [15]. Increasing evidence suggests OS plays a major role in the pathogenesis of both types of DM [16]. MDA, a toxic molecule produced by ROS, is induced by lipid peroxidation of the cellular membrane, and its level can represent the degree of lipid peroxidation and OS. SOD is an important enzyme that removes the superoxide radicals, and its activity can be inhibited by excess amount of ROS [17]. Moreover, the hyperglycemia environment could cause the accumulation of AGEs and their receptor RAGE, to impair vascular endothelium function and activate NADPH-induced OS [18–21]. Previous study has verified the importance of OS in the DMED rat model, and the major sources of ROS in the vessel wall are the vascular NADPH oxidases including subunits such as NOX2, p47^phox^, and p67^phox^ subunits [22]. In consistent with the studies above, our data suggested a higher MDA level and a lower SOD activity under DM environment, while hKLK1 could improve these pathological changes through inhibition of RAGE and NADPH oxidases. Additionally, *in vitro* experiments verified the role of OS under high-glucose condition and the protective role of hKLK1 in CSMC by ROS and OS-related proteins level detection. However, HOE140, the hKLK1 pathway inhibitor, antagonized the protective effect of hKLK1 and reactivated OS in CSMC. Therefore, we conclude that excessive OS is indeed involved in the DMED, and hKLK1 can inhibit it.

Overactivated OS could impair erectile function through induction of penile functional cells apoptosis under many conditions including aging, hyperlipidemia, and DM [9, 23, 24]. Our data verified the inhibitory effect of hKLK1 on cells apoptosis through detection of endothelium, smooth muscle contents, and apoptotic percentage in tissue slides and further elucidated the mechanisms of reducing over expressions of Caspase3, cleaved Caspase3, and elevated ratio of Bax/Bcl-2. However, the use of HOE140 in TGDMH group eliminated these benefits of hKLK1. *In vitro* experiments also supported the inhibitory role of hKLK1 in CSMC apoptosis under high-glucose condition.

Cavernous fibrosis is a serious morphological change to cause refractory ED [9, 25–28]. Many studies have demonstrated the existence of positive feedback regulation between ROS and TGF*β*1, and TGF*β*1-dependent fibrotic changes occur in penile tissue of ED patients regardless of etiology [29–31]. Increased TGF*β*1 activity could activate smad/CTGF signaling and increase collagen deposition in the penis, which resulted in cavernous fibrosis and the strong restriction of erection [27]. However, numerous studies demonstrated that *KLK* gene delivery could reduce renal, cardiac, or cavernous fibrosis in rats through its suppression of TGF*β*1 signaling [9, 32, 33]. Our data initially confirmed that hKLK1 could reduce TGF*β*1 level in penile tissue of DM rats and then inhibit the smad/CTGF/collagen IV signaling activity to repress collagen deposition. This might improve penile compliance, veno-occlusive mechanism, and cavernous response to NO/cGMP in DM rats.

The production and release of NO by nonadrenergic and noncholinergic nerves and EC have been shown to be the major mediator to induce cavernous smooth muscle relaxation via activating NO/cGMP signal pathway [34, 35]. DM-induced OS can inhibit the activity of eNOS and the formation of endogenous NO, then reduce cGMP level in CSMC [36]. Previous study reported KKS could activate PI3K/AKT/eNOS signaling in cardiovascular system [37]. Our data showed that hKLK1 could upregulate the expression of PI3K, AKT, and eNOS in corpus cavernosum of DM rats. Meanwhile, we also found hKLK1 could increase the activity of eNOS by regulation of their key phosphorylation sites [38], which in common raised NO and cGMP levels. In addition, *in vitro* experiments showed hKLK1 could elevate cGMP level in both physiological and high-glucose conditions, while this effect can be counteracted by the specific inhibitors of B_2_R, PI3K, and eNOS. Together, these findings indicate hKLK1 has the ability to activate the NO/cGMP pathway through regulation of PI3K/AKT/eNOS signaling.

Ca^2+^ is a vital ion to regulate relaxation and contraction of smooth muscle, which is considered as the most important ion involved in penile erection. ICa-L, accessing Ca^2+^ from extracellular fluid into cytoplasm, is a major ion channel that causes cavernous smooth muscle contraction. Previous research demonstrated that activation of the NO/cGMP pathway could close the ICa-L and open potassium channels on cytomembrane to induce the decrease of cytosolic Ca^2+^ concentration and cell hyperpolarization, generating tumescence of the penis [39]. Our data showed hKLK1 could finally impact ICa-L expression to regulate Ca^2+^ influx through PI3K/AKT/eNOS signaling to promote penile erection in DM rats.

Although our data proves the protective effects of hKLK1 on erectile function of DM rats *in vivo* and *in vitro*, some limitations remain. First is the replacement of aorta EC for cavernous EC, though they have similar properties. Meanwhile, we are still trying to explain why hKLK1 had unexpected ability to reduce the expression of PDE5 in corpus cavernosum. Lastly, insulin was not involved in this study to determine its therapeutic effectiveness, given previous studies showed limited or no effect on DMED.

## 5. Conclusions

hKLK1 preserves erectile function of DM rats through its antitissue OS, apoptosis, fibrosis effects, and activation of the PI3K/AKT/eNOS/cGMP pathway in the penis. Moreover, hKLK1 promotes relaxation and prevents high glucose-induced injuries of CSMC mediated by EC-CSMC crosstalk. Considering the high homology of the *hKLK1* gene in the human race, it can be expected as a promising target due to its better effectiveness and safety in human.

## Figures and Tables

**Table 1:** Primers used in conventional PCR and real-time RT-PCR.

Genes	Primer sequences	Usage (PCR)
*hKLK1*	F: 5′-GTCCAGAAGGTGACAGACTTCAT-3′ R: 5′-GTCCTCGATCCACTTCACATAAG-3′	Conventional
	F: 5′-CTCACAGCTGCTCATTGCATC-3′ R: 5′-GCTCTCACTGACATGAACAAACTGG-3′	Real time

*rKLK1*	F: 5′-CCCACACACAGATGGTGACAGA-3′ R: 5′-CCTTGAAGCACACCATCACAGAG-3′	Real time

*β-Actin*	F:5′-AAGAGCTATGAGCTGCCTGA-3′ R: 5′-TACGGATGTCAACGTCACAC-3′	Conventional and real time

PCR = polymerase chain reaction; hKLK1 = human tissue kallikrein 1; rKLK1 = rat tissue kallikrein 1.

**Table 2:** Body weight and fasting blood glucose level before and after treatment.

Groups	Initial body weight (g)	Final body weight (g)	Initial FBG (mmol/L)	Final FBG (mmol/L)
WTR	225.75 ± 5.42	495.75 ± 28.22	5.40 ± 0.19	5.61 ± 0.22
TGR	233.25 ± 4.08	462.50 ± 25.20	5.46 ± 0.15	5.63 ± 0.16
WTDM	226.13 ± 5.81	213.13 ± 19.51^∗^	5.41 ± 0.22	29.99 ± 1.46^∗^
TGDM	232.50 ± 3.76	234.13 ± 13.98^∗^	5.35 ± 0.20	30.86 ± 1.26^∗^
TGDMH	227.75 ± 4.05	222.88 ± 14.19^∗^	5.39 ± 0.22	31.04 ± 0.82^∗^

^∗^ *P* < 0.001 compared with WTR group. WTR: wild-type rats; TGR: transgenic rats; WTDM: diabetic wild-type rats; TGDM: diabetic transgenic rats; TGDMH: diabetic transgenic rats administrated with HOE140; FBG: fasting blood glucose.

## Data Availability

The data of the materials and methods and results to support the conclusions are included in this article. If any other data are needed, please contact the corresponding author.
